# The association between polymorphism of the long noncoding RNA, *Plasmacytoma variant translocation 1*, and the risk of gastric cancer

**DOI:** 10.1097/MD.0000000000027773

**Published:** 2021-12-03

**Authors:** Jae Ho Park, Eun-Heui Jin, Jang Hee Hong, Sang-Il Lee, Jae Kyu Sung

**Affiliations:** a Department of Internal Medicine, Chungnam National University Hospital, Chungnam National University College of Medicine, Daejeon, Republic of Korea; b Translational Immunology Institute, Chungnam National University College of Medicine, Daejeon, Republic of Korea; c Department of Pharmacology, Chungnam National University College of Medicine, Daejeon, Republic of Korea; d Clinical Trials Center, Chungnam National University Hospital, Daejeon, Republic of Korea; e Department of Surgery, Chungnam National University Hospital, Chungnam National University College of Medicine, Daejeon, Republic of Korea.

**Keywords:** *Plasmacytoma variant translocation 1*, polymorphism, single nucleotide, stomach neoplasms

## Abstract

Genetic polymorphisms of plasmacytoma variant translocation 1 can affect various tumors including gastro-intestinal, sexual hormone sensitive cancers and lymphoma. Accumulated evidence have shown that plasmacytoma variant translocation 1 acts as an oncogene and tumor suppressor in various cancers. In fact, the rs13255292 and rs2608053 single nucleotide polymorphisms of plasmacytoma variant translocation 1are known to affect lymphoma; however, their effects on gastric cancer are primarily unknown. In this study, we evaluated the association between these plasmacytoma variant translocation 1 polymorphisms and the risk of gastric cancer.

In the present study, 462 patients diagnosed with gastric cancer and 377 cancer-free controls were enrolled. The TaqMan genotyping assay was used to analyze the association between rs13255292 and rs2608053 single nucleotide polymorphisms and the risk of gastric cancer.

The rs2608053 dominant model (CT + TT) was associated with a decreased risk of gastric cancer in T3 + T4 (odds ratio [OR] = 0.61, confidence interval (CI) = 0.41 – 0.92, *P* = .019), and stage III Gastric cancer subgroups (OR = 0.59, 95% CI = 0.38 – 0.91, *P* = .017) compared to the CC genotype. When stratified analysis by sex was carried out, the rs13255292 dominant model (CT + TT) had a significant association with an increased risk of gastric cancer in the female negative lymph node metastasis gastric cancer subgroup, compared to the CC genotype (OR = 1.96, 95% CI = 1.16 – 3.30, *P* = .012). The recessive model (TT) of rs13255292 was associated with an increased risk of gastric cancer in the male T3 + T4 gastric cancer subgroups compared to the CC + CT genotype (OR = 3.82, 95% CI = 1.02 – 14.33, *P* = .047). The dominant model (CT + TT) of rs2608053 was related to a decreased risk of gastric cancer in male T3 + T4 (OR = 0.57, 95% CI = 0.33 – 0.98, *P* = .042) and stage III gastric cancer subgroups (OR = 0.49, 95% CI = 0.27 – 0.89, *P* = .020) compared to the CC genotype.

The rs13255292 and rs2608053 single nucleotide polymorphisms in plasmacytoma variant translocation 1 may contribute to susceptibility of gastric cancer. Further studies with more subjects and different ethnic groups are needed to validate our results.

## 1 Introduction

Gastric cancer (GC) is the second most common cancer worldwide and has the highest mortality rate among cancers. In 2018, the most common cancer in South Korea was GC, with incidence and mortality rates of 57.1% and 14.9 per 100,000 people, respectively. The 5-year survival rate of GC is 96.9% for localized cases, 61.7% for regional cases, and 5.9% for distant metastasis cases.^[1]^ Despite recent advances in diagnosis, treatment, and chemotherapy, the prognosis of advanced-stage GC remains poor. Therefore, it is important to identify markers that influence GC susceptibility. Long non-coding RNAs (lncRNAs) are transcribed RNA molecules that are greater than 200 nucleotides in length. The genetic polymorphism of lncRNAs has been demonstrated to influence the expression of tumor characteristics by carrying out molecular functions that influence the development and differentiation of cells or tissues.^[2]^ Plasmacytoma variant translocation 1 (*PVT1*) is a lncRNA located on chromosome 8q24. *PVT1* that is 55 kb distal to the *C-MYC* gene functions as an oncogene and has been found in several tumors. *PVT1* encodes multiple miRNAs (miR-1204, miR-1205, miR1206, miR1207-5p, miR-1207-3p, miR-1208,^[3]^ miR-152,^[4,5]^ and miR-186^[6]^) and has been reported to exhibit oncogenic properties.^[3–5,7–9]^ Polymorphisms of *PVT1* have been shown to affect familial predisposition by acting as a genetic risk factor in lymphoma.^[10]^ The accumulation of *PVT1* has been reported in esophageal,^[11]^ gastric,^[5,6,12–18]^ colorectal,^[19]^ lung,^[20]^ ovarian and breast cancer,^[21]^ lymphoma,^[10,22–24]^ prostate^[25]^ and pancreatic^[26]^ cancer, and hepatocellular carcinoma.^[27]^

According to an analysis of the Progenetix copy number database, 98.7% of tumors display increased copy number at 8q24 and increased copy number of the *PVT1* and *MYC* genes. This characteristic was demonstrated by a reduction in tumorigenic potency, especially when *PVT1* was removed from colon cancer cells.^[28]^*PVT1* has been reported to be associated with the oncogenic and tumor suppressor pathways in GC,^[29]^ such as c-MYC,^[15]^ FOMX1,^[9]^ NOP2,^[27]^ CCNB1, AURKB, STAT3/VEGFA,^[16]^ and SKP2. rs13255292 and rs2608053 single nucleotide polymorphisms (SNPs) in *PVT1* have been reported to affect lymphoma.^[22,30]^ However, the effects of rs13255292 and rs2608053 SNPs in *PVT1* on GC risk are still unknown. We hypothesized that *PVT1* SNPs might affect the genetic susceptibility to GC. Therefore, we performed a case-control study to investigate the association between the SNPs in *PVT1* and the risk of GC in the Korean population.

## 2 Materials and methods

### 2.1 Ethics statement

The present study was conducted in accordance with the Declaration of Helsinki and was reviewed and approved by the Ethics Committee of the Institutional Review Board of Chungnam National University Hospital on July 23, 2017. Informed consent was provided by all subjects upon enrolment (IRB file No. CNUH 2017-07-023).

### 2.2 Patients and control

A total of 462 patients diagnosed with GC and 377 cancer-free control subjects were enrolled at the Chungnam National University Hospital. Blood samples were provided by the Chungnam National University Hospital Biobank, a member of the National Biobank that is supported and audited by the Ministry of Health and Welfare in South Korea. All blood sample donors provided written informed consent. GC patients were recruited from the outpatient clinic of Chungnam National University Hospital, and healthy controls without a history of cancer were randomly selected from the Chungnam National University Hospital's Health Screening Center. The role of SNP was evaluated by comparing the allele frequency of lncRNAs in the tumor group to that in healthy controls.

### 2.3 DNA isolation and genotyping

Genomic DNA was isolated from peripheral blood using the QIAamp DNA Blood Mini Kit (Qiagen GmbH, Hilden, Germany), according to the manufacturer's instructions. Based on previous studies,^[10,12,14,17,18,23,24,31]^ two SNPs (rs13255292 and rs2608053) in *PVT1* were selected. Genotyping was conducted using the Applied Biosystems TaqMan SNP Genotyping Assay and the StepOnePlus Real-Time PCR System (Applied Biosystems, Foster City, CA).

### 2.4 Statistical analysis

The chi-square test was used to assess the Hardy-Weinberg equilibrium for each SNP in the control group. A pair-wise comparison of the biallelic loci was employed to analyze the linkage disequilibrium using Haploview software (version 4.0; Broad Institute, Cambridge, MA).

The differences in age, sex, and other factors between GC patients and controls were calculated using the *χ*^2^ test and the Mann-Whitney *U*-test. The association between GC and these factors was analyzed using a dominant and recessive genetic model. The binary logistic regression method was used to analyze the association between genetic factors and clinical features (age, gender, tumor differentiation, histological type, T classification, lymph node metastasis [LNM], tumor stage, and lympho-vascular invasion). The results are presented as odds ratios (ORs) and 95% confidence intervals (CIs). The association analysis was adjusted for age and sex, which were included as covariates in the model. Statistical significance was set at *P* < .05. Statistical analyses were performed using SPSS software version 22 for Windows (SPSS Inc., Chicago, IL).

## 3 Results

### 3.1 Patient characteristics

The clinical characteristics of the 462 GC patients and the 377 control subjects are shown in Table 1. Significant differences in age and sex distribution were identified between the two groups (*P* < .001). The mean age of patients with GC was 65.2 ± 11.0 while that of controls was 56.1 ± 10.9 years. Male predominance (70.1%) was observed in the GC group, while female predominance (68.2%) was observed in the control group. Among the 462 GC patients, the most common pathologic type was the intestinal type (56.1%). However, when classified according to the AJCC 8th edition staging system, stage I (59.1%) was identified to be the most common. Regarding the tumor characteristics, factors, such as differentiation, histological type, T stage classification, LNM, and TNM staging were evaluated.

**Table 1:** Baseline characteristics.

Variables		Gastric cancer	Controls	*P*
Age (yr) (mean ± SD)		462 (65.2 ± 11.0)	377 (56.1 ± 10.9)	< .001^†^
	<60	197 (52.2 ± 5.8)	195 (45.8 ± 5.5)	.009^‡^
	≥60	265 (71.4 ± 6.4)	182 (64.4 ± 4.4)	
Sex (%)	Male	324 (70.1)	120 (31.8)	<.001^‡^
	Female	138 (29.9)	257 (68.2)	
Tumor differentiation				
	Differentiated	199 (43.1)		
	Undifferentiated	223 (48.2)		
	Missing	40 (8.7)		
Histological type (%)				
	Intestinal	259 (56.1)		
	Diffuse	148 (32.0)		
	Mixed	55 (11.9)		
T classification (%)^∗^				
	T1	233 (50.4)		
	T2	67 (14.5)		
	T3	16 (3.5)		
	T4	146 (31.6)		
Lymph node metastasis (%)				
	Negative	283 (61.3)		
	Positive	179 (38.7)		
Tumor stage (%)^∗^				
	I (A + B)	273 (59.1)		
	II (A + B)	55 (11.9)		
	III (A + B + C)	134 (29.0)		

### 3.2 Association between *PVT1* SNPs and GC risk

We selected two *PVT1* SNPs (rs13255292 and rs2608053) that have previously been associated with several cancers, such as lymphoma,^[22,30]^ ovarian cancer.^[32]^ The genotype frequencies of the rs13255292 and rs2608053 SNPs were not found to deviate from Hardy- Weinberg equilibrium in the control group (*P* = .716 and *P* = .935, respectively). The genotypes of the rs13255292 and rs2608053 SNPs in *PVT1* are shown in Table 2. No significant association was found between these SNPs and GC risk (Table 2).

**Table 2:** Genotype and allele frequencies for *PVT1* two SNPs among subject and their association with GC risk.

SNP	Genotype	GC, N (%)	Control, N (%)	AOR (95% CI)	*P* ^∗^
rs13255292	CC	292 (63.2)	250 (66.3)	1.00 (ref.)	
	CT	146 (31.6)	111 (29.5)	1.09 (0.78–1.50)	.622
	TT	24 (5.2)	16 (4.2)	1.30 (0.64–2.64)	.468
	C	730 (79.0)	611 (81.0)	1.00 (ref.)	
	T	194 (21.0)	143 (19.0)	1.12 (0.86–1.45)	.415
	Dominant model				
	CC	292 (63.2)	250 (66.3)	1.00 (ref.)	
	CT + TT	170 (36.8)	127 (33.7)	1.11 (0.82–1.52)	.502
	Recessive model				
	CC + CT	438 (94.8)	361 (95.8)	1.00 (ref.)	
	TT	24 (5.2)	16 (4.2)	1.27 (0.63–2.55)	.509
			P_HWE_ = 0.716		
rs2608053	CC	262 (56.7)	202 (53.6)	1.00 (ref.)	
	CT	157 (34.0)	146 (38.7)	0.88 (0.64–1.20)	.418
	TT	43 (9.3)	29 (7.7)	0.93 (0.53–1.60)	.780
	C	681 (73.7)	550 (72.9)	1.00 (ref.)	
	T	243 (26.3)	204 (27.1)	0.92 (0.73–1.17)	.499
	Dominant model				
	CC	262 (56.7)	202 (53.6)	1.00 (ref.)	
	CT + TT	200 (43.3)	175 (46.4)	0.89 (0.66–1.19)	.426
	Recessive model				
	CC + CT	419 (90.7)	348 (92.3)	1.00 (ref.)	
	TT	43 (9.3)	29 (7.7)	0.97 (0.57–1.66)	.922
			P_HWE_ = 0.935		

### 3.3 Stratified analysis of the rs13255292 and rs2608053 SNPs

We performed a stratified analysis to determine the relationship between *PVT1* SNPs and the GC risk in patients with GC and controls according to various clinical factors, including age, sex, tumor differentiation, histological type, T classification, LNM, and tumor stage (Table 3, Supplementary Digital Content Table S1).

**Table 3:** Stratified analysis of rs2608053 SNP of *PVT1* in GC patients and controls by clinical features.

		Dominant model (CC/CT + TT)				Recessive model (CC + CT/TT)			
Features		CON, N	GC, N	AOR (95% CI)	*P* ^a^	CON, N	GC, N	AOR (95% CI)	*P* ^a^
Age	<60	87 (44.4)	75 (38.1)	0.80 (0.50–1.28)	.345	13 (6.6)	11 (5.6)	0.74 (0.28–1.95)	.546
	≥60	88 (48.6)	125 (47.2)	1.02 (0.68–1.54)	.916	16 (8.8)	32 (12.1)	1.18 (0.60–2.31))	.637
Sex	M	53 (43.8)	141 (43.5)	0.99 (0.65–1.52)	.976	14 (11.6)	32 (9.9)	0.85 (0.44–1.66)	.636
	F	122 (47.7)	59 (42.8)	0.80 (0.52–1.22)	.291	15 (5.9)	11 (8.0)	1.23 (0.54–2.81)	.620
T	T1 + T2	175 (46.4)	145 (48.3)	1.08 (0.78–1.51)	.644	29 (7.7)	30 (10.0)	1.04 (0.58–1.87)	.887
	T3 + T4	175 (46.4)	55 (34.0)	0.61 (0.41–0.92)	.019^∗^	29 (7.7)	13 (8.1)	0.76 (0.36–1.57)	.455
LNM	Positive	175 (46.4)	67 (37.4)	0.74 (0.50–1.09)	.123	29 (7.7)	18 (10.1)	1.09 (0.57–2.12)	.790
	Negative	175 (46.4)	133 (47.0)	1.02 (0.72–1.42)	.933	29 (7.7)	25 (8.8)	0.83 (0.45–1.53)	.554
Stage	I + II	175 (46.4)	156 (47.6)	1.04 (0.75–1.43)	.833	29 (7.7)	31 (9.5)	0.96 (0.54–1.71)	.891
	III	175 (46.4)	44 (32.8)	0.59 (0.38–0.91)	.017^∗^	29 (7.7)	12 (8.9)	0.84 (0.39–1.78)	.648
Histology	Intestinal	175 (46.4)	114 (44.0)	0.91 (0.63–1.30)	.602	29 (7.7)	28 (10.8)	0.97 (0.52–1.79)	.922
	Diffuse	175 (46.4)	64 (43.2)	0.91 (0.61–1.36)	.652	29 (7.7)	12 (8.1)	0.97 (0.47–2.01)	.929

After adjusting for age and sex, the rs2608053 dominant model (CT + TT) showed a significant association with a decreased risk of GC in the T3 + T4 and stage III subgroups compared to the CC genotype (OR = 0.61, 95% CI = 0.41 – 0.92, *P* = .019 and OR = 0.59, 95% CI = 0.38 – 0.91, *P* = .017, respectively) (Table 3). When a stratified analysis by sex was carried out, the rs13255292 dominant model (CT + TT) in the female LNM-negative subgroup was significantly associated with an increased risk of GC compared to the CC genotype (OR = 1.96, 95% CI = 1.16 – 3.30, *P* = .012) (Table 4). The recessive model (TT) of rs13255292 was associated with an increased risk of GC in the male T3 + T4 subgroup compared to the CC + CT genotype (OR = 3.82, 95% CI = 1.02 – 14.33, *P* = .047) (Table 4). The dominant model (CT + TT) of rs2608053 was associated with a decreased risk of GC in male T3 + T4 and stage III subgroups compared to the CC genotype (OR = 0.57, 95% CI = 0.33 – 0.98, *P* = .042, and OR = 0.49, 95% CI = 0.27 – 0.89, *P* = .020, respectively) (Table 5). When the stratified analysis by age was performed, no significant association was found between rs13255292 and rs2608053 SNPs and GC risk (Supplementary Digital Content Table 2, 3).

**Table 4:** Stratified analysis of rs13255292 SNP of *PVT1* in GC patients and controls by sex and other clinical features.

			Dominant model (CC/CT + TT)				Recessive model (CC + CT/TT)			
Sex	Features		CON, N	GC, N	AOR (95% CI)	*P* ^a^	CON, N	GC, N	AOR (95% CI)	*P* ^a^
Male	Age	<60	19 (42.2)	55 (39.0)	0.81 (0.40–1.62)	.542	1 (2.2)	6 (4.3)	1.86 (0.22–16.15)	.572
		≥60	26 (34.7)	65 (35.5)	1.09 (0.62–1.93)	.770	2 (2.7)	12 (6.5)	2.59 (0.56–11.95)	.223
	T	T1 + T2	45 (37.5)	82 (37.8)	0.97 (0.61–1.55)	.912	3 (2.5)	8 (3.7)	1.43 (0.37–5.54)	.602
		T3 + T4	45 (37.5)	38 (35.5)	0.95 (0.55–1.65)	.864	3 (2.5)	10 (9.3)	3.82 (1.02–14.33)	.047^∗^
	LNM	Positive	45 (37.5)	43 (36.1)	0.98 (0.58–1.67)	.943	3 (2.5)	8 (6.7)	2.59 (0.67–10.10)	.169
		Negative	45 (37.5)	77 (37.6)	0.96 (0.60–1.54)	.875	3 (2.5)	10 (4.9)	1.92 (0.52–7.16)	.331
	Stage	I + II	45 (37.5)	88 (37.3)	0.95 (0.60–1.51)	.841	3 (2.5)	10 (4.2)	1.66 (0.45–6.17)	.451
		III	45 (37.5)	32 (36.4)	1.02 (0.57–1.83)	.947	3 (2.5)	8 (9.1)	3.51 (0.89–13.84)	.073
	Histology	Intestinal	45 (37.5)	75 (37.7)	1.02 (0.64–1.64)	.934	3 (2.5)	15 (7.5)	3.18 (0.90–11.23)	.072
		Diffuse	45 (37.5)	27 (31.0)	0.71 (0.39–1.29)	.266	3 (2.5)	2 (2.3)	1.06 (0.17–6.56)	.954
Female	Age	<60	53 (35.3)	23 (41.1)	1.32 (0.69–2.53)	.397	9 (6.0)	1 (1.8)	0.36 (0.04–2.92)	.337
		≥60	29 (27.1)	27 (32.9)	1.05 (0.52–2.13)	.886	4 (3.7)	5 (6.1)	1.62 (0.39–6.79)	.509
	T	T1 + T2	82 (31.9)	36 (43.4)	1.65 (0.99–2.75)	.054	13 (5.1)	4 (4.8)	0.91 (0.29–2.88)	.872
		T3 + T4	82 (31.9)	14 (25.5)	0.79 (0.40–1.56)	.492	13 (5.1)	2 (3.6)	0.73 (0.15–3.50)	.698
	LNM	Positive	82 (31.9)	13 (21.7)	0.63 (0.32–1.24)	.181	13 (5.1)	2 (3.3)	0.66 (0.14–3.06)	.591
		Negative	82 (31.9)	37 (47.4)	1.96 (1.16–3.30)	.012^∗^	13 (5.1)	4 (5.1)	0.97 (0.30–3.07)	.952
	Stage	I + II	82 (31.9)	39 (42.4)	1.61 (0.98–2.65)	.060	13 (5.1)	4 (4.3)	0.81 (0.26–2.58)	.726
		III	82 (31.9)	11 (23.9)	0.71 (0.34–1.48)	.358	13 (5.1)	2 (4.3)	0.86 (0.18–4.08)	.852
	Histology	Intestinal	82 (31.9)	19 (31.7)	0.99 (0.52–1.88)	.973	13 (5.1)	3 (5.0)	1.01 (0.26–3.85)	.993
		Diffuse	82 (31.9)	26 (42.6)	1.20 (0.67–2.15)	.546	13 (5.1)	5 (8.2)	0.64 (0.14–2.91)	.562

**Table 5:** Stratified analysis of rs2608053 SNP of *PVT1* in GC patients and control by sex and other clinical features.

			Dominant model (CC/CT + TT)				Recessive model (CC + CT/TT)			
Sex	Features		CON, N	GC, N	AOR (95% CI)	*P* ^a^	CON, N	GC, N	AOR (95% CI)	*P* ^a^
Male	Age	<60	21 (45.7)	51 (36.2)	0.68 (0.35–1.35)	.272	5 (10.9)	8 (5.7)	0.54 (0.16–1.75)	.301
		≥60	32 (42.7)	90 (49.2)	1.40 (0.81–2.43)	.234	9 (12.0)	24 (13.1)	1.10 (0.48–2.51)	.823
	T	T1 + T2	53 (43.8)	108 (49.8)	1.33 (0.84–2.08)	.221	14 (11.6)	24 (11.1)	1.02 (0.50–2.06)	.962
		T3 + T4	53 (43.8)	33 (30.8)	0.57 (0.33–0.98)	.042^∗^	14 (11.6)	8 (7.5)	0.57 (0.23–0.98)	.226
	LNM	Positive	53 (43.8)	41 (34.5)	0.68 (0.40–1.14)	.143	14 (11.6)	10 (8.4)	0.67 (0.28–1.57)	.353
		Negative	53 (43.8)	100 (48.8)	1.29 (0.81–2.03)	.281	14 (11.6)	22 (10.7)	1.00 (0.49–2.06)	.997
	Stage	I + II	53 (43.8)	116 (49.2)	1.29 (0.83–2.01)	.261	14 (11.6)	25 (10.6)	0.96 (0.48–1.94)	.919
		III	53 (43.8)	25 (28.4)	0.49 (0.27–0.89)	.020^∗^	14 (11.6)	7 (7.9)	0.57 (0.21–1.50)	.252
	Histology	Intestinal	53 (43.8)	87 (43.7)	0.99 (0.63–1.56)	.958	14 (11.6)	23 (11.6)	0.98 (0.48–1.99)	.945
		Diffuse	53 (43.8)	38 (43.7)	1.02 (0.58–1.79)	.946	14 (11.6)	7 (8.1)	0.72 (0.27–1.89)	.503
Female	Age	<60	66 (44.0)	24 (42.9)	0.91 (0.48–1.72)	.765	8 (5.3)	3 (5.4)	1.19 (0.29–4.83)	.813
		≥60	56 (52.8)	35 (42.7)	0.62 (0.32–1.18)	.147	7 (6.6)	8 (9.8)	1.24 (0.39–3.96)	.719
	T	T1 + T2	122 (47.7)	37 (44.6)	0.87 (0.53–1.44)	.594	15 (5.9)	6 (7.2)	1.19 (0.44–3.18)	.734
		T3 + T4	122 (47.7)	22 (40.0)	0.66 (0.36–1.23)	.189	15 (5.9)	5 (9.1)	1.22 (0.40–3.70)	.732
	LNM	Positive	122 (47.7)	26 (43.3)	0.80 (0.45–1.44)	.461	15 (5.9)	8 (13.3)	2.05 (0.80–5.26)	.136
		Negative	122 (47.7)	33 (42.3)	0.78 (0.46–1.31)	.342	15 (5.9)	3 (3.8)	0.60 (0.17–2.15)	.432
	Stage	I + II	122 (47.7)	40 (43.5)	0.83 (0.51–1.34)	.440	15 (5.9)	6 (6.5)	1.05 (0.39–2.83)	.918
		III	122 (47.7)	19 (41.3)	0.72 (0.38–1.39)	.327	15 (5.9)	5 (10.9)	1.53 (0.50–4.61)	.455
	Histology	Intestinal	122 (47.7)	27 (45.0)	0.83 (0.46–1.51)	.545	15 (5.9)	5 (8.3)	1.16 (0.38–3.54)	.796
		Diffuse	122 (47.7)	26 (42.6)	0.82 (0.47–1.44)	.486	15 (5.9)	5 (8.2)	1.46 (0.51–4.19)	.484

## 4 Discussion

The aim of the present study was to evaluate the association between SNPs (rs13255292 and rs2608053) in *PVT1* and the risk of GC in the Korean population. Although there was no significant association between rs13255292 and rs2608053 in *PVT1* and the overall risk of GC, rs13255292 dominant model (CT + TT) and recessive model (TT) were significantly associated with higher risk of GC in the female negative LNM and male T3 + T4 GC subgroup respectively, after stratified analysis. To our knowledge, this is the first study to investigate the relationship between rs13255292 and rs2608053 SNPs in *PVT1* and GC. Some studies have reported an association between *PVT1* SNPs and cancer. The T allele of rs13255292 has been found to increase the risk of diffuse large B-cell lymphoma at stages 1, 2, and 3 by 1.19-, 1.30-, and 1.22-fold, respectively.^[30]^ The T allele of rs13255292 has also been shown to reduce the risk of ovarian cancer in women taking oral contraceptive pill.^[32]^ Further, the recessive model (TT) of rs13255292 has been shown to decrease the risk of glioma in the male.^[33]^ A previous study investigated the association between the genetic polymorphisms of *PVT1* and the risk of lung cancer.^[34]^ However, no statistically significant relationship was found between rs2608053 polymorphisms in *PVT1* and the risk of lung cancer in the overall population. Subjects with both the AG + AA rs2608053 genotype and smoking exposure had a higher risk of lung cancer and non-small cell lung cancer than the GG genotype with non-smoking exposure.^[34]^

Several studies have shown that lncRNA SNPs are associated with tumor characteristics, have functional effects on gene expression, and serve as a potential prognostic biomarker. Recently, a case-control study to evaluate the association between haplotype-tagging SNPs of Hox transcript antisense intergenic RNA (*HOTAIR*) and the susceptibility to gastric cardia cancer has been performed. It found that T allele of rs12826786 was associated with TNM stage and rs12826786 SNP had a genotype-specific influence on *HOTAIR* expression. High *HOTAIR* expression was related to poor survival. This study indicated the functional effect of the susceptibility rs12826786 SNP on *HOTAIR* expression.^[35]^

Ma et al genotyped the 940 Chinese GC patients who underwent surgery to evalauate the association between two SNPs (e.g. rs10505477 and rs1562430) in the intron of Cancer Susceptibility Candidate 8 and survival of GC.^[36]^ They found that GC patients with rs10505477 GG genotype survived for a longer time compared with those carrying the GA and AA. This prognostic risk effect was more significant among patients with tumor size ≤ 5 cm, diffuse-type GC, LNM, no distant metastasis, and TNM stage III and IV. This study suggested SNP rs10505477 in *CASC8* may be a potential marker to predict the survival of GC in Chinese populations. Additionally, Hong et al showed a relationship between lncRNA prostate cancer non-coding RNA1 SNPs and risk of GC in LNM-positive and stage III subgroups.^[13]^ Although our study had a different purpose from the studies mentioned above, we plan to do more research on the functional role of SNPs and their relationship to cancer characteristics in the future.

Sex is one of the most important factors influencing various diseases, including cancer. Substantial studies have shown that there are significant differences between male and female subpopulations in terms of cancer incidence, prognosis, mortality, and treatment response.^[37]^ Although we were not sure whether a genetic variation affect GC formation differentially in sex, we performed stratified analysis based on these statistical data expecting statistical difference in GC subgroups including sex. As a result, we detected significant differences between *PVT1* polymorphisms and LNM and tumor stage in male or female GC subgroup. Further studies are required to validate our findings.

This study had some limitations. First, the sample size was relatively small, which may have resulted in a weak statistical power. Second, we failed to study the association between the SNPs and other clinical features, such as *Helicobacter pylori* infection, smoking, drinking, diet and family history of cancer due to the lack of data from the GC and control groups. Third, the subjects in this study were from a specific ethnic group. Fourth, there was a difference on age and sex distribution between cases and controls. Therefore, we used unconditional logistic regression in the analysis of the association. Further studies are thus required to validate our results in different ethnic groups

In conclusion, our findings suggest that the rs13255292 and rs2608053 SNPs in *PVT1* may be associated with GC risk in certain GC subgroups characterized by LNM, tumor stage, and sex. However, further studies with different ethnic groups are required to validate these findings.

## Acknowledgments

All authors met the authorship criteria set forth by the International Committee for Medical Journal Editors and retained full control of the manuscript content.

## Author contributions

**Conceptualization:** Jae Kyu Sung.

**Data curation:** Jae Ho Park and Eun-Heui Jin.

**Formal analysis:** Jae Ho Park and Eun-Heui Jin.

**Investigation:** Sang-Il Lee.

**Methodology:** Jang Hee Hong.

**Supervision:** Jae Kyu Sung.

**Writing – original draft:** Jae Ho Park.

**Writing – review & editing:** Jae Kyu Sung and Eun-Heui Jin.

## Supplementary Material

Supplemental Digital Content

## Supplementary Material

Supplemental Digital Content

## Supplementary Material

Supplemental Digital Content
