# Supplementary material for: The association between polymorphism of the long noncoding RNA, Plasmacytoma variant translocation 1, and the risk of gastric cancer
Source: Medicine (Baltimore). 2021 Dec 3;100(48):e27773. doi: 10.1097/MD.0000000000027773 (PMC9191314; doi:10.1097/MD.0000000000027773)
Supplement: Supplemental Digital Content [file medi-100-e27773-s001.pdf]

Table S1 Stratified analysis of rs13255292 SNP of *PVT1* in GC patients and controls by clinical features

Features		Dominant model (CC/CT+TT)				Recessive model (CC+CT/TT)			
		CON, N	GC, N	AOR (95% CI)	P^{a}	CON, N	GC, N	AOR (95% CI)	P^{a}
Age	<60	72 (36.9)	78 (39.6)	1.04 (0.65-1.68)	0.869	10 (5.1)	7 (3.6)	0.77 (0.24-2.52)	0.670
	≥60	55 (30.2)	92 (34.7)	1.13 (0.73-1.75)	0.581	6 (3.3)	17 (6.4)	2.03 (0.74-5.54)	0.167
Sex	M	45 (37.5)	120 (37.0)	0.97 (0.63-1.50)	0.887	3 (2.5)	18 (5.6)	2.31 (0.67-8.00)	0.186
	F	82 (31.9)	50 (36.2)	1.25 (0.80-1.95)	0.320	13 (5.1)	6 (4.3)	0.81 (0.30-2.20)	0.674
T	T1+T2	127 (33.7)	118 (39.3)	1.26 (0.89-1.77)	0.194	16 (4.2)	12 (4.0)	1.15 (0.50-2.65)	0.742
	T3+T4	127 (33.7)	52 (32.1)	0.89 (0.58-1.36)	0.581	16 (4.2)	12 (7.4)	1.75 (0.75-4.07)	0.193
LNM	Positive	127 (33.7)	56 (31.3)	0.83 (0.55-1.25)	0.366	16 (4.2)	10 (5.6)	1.31 (0.54-3.18)	0.544
	Negative	127 (33.7)	114 (40.3)	1.35 (0.95-1.91)	0.094	16 (4.2)	14 (4.9)	1.37 (0.61-3.06)	0.445
Stage	I+II	127 (33.7)	127 (38.7)	1.23 (0.88-1.73)	0.224	16 (4.2)	14 (4.3)	1.16 (0.52-2.59)	0.711
	III	127 (33.7)	43 (32.1)	0.89 (0.56-1.39)	0.600	16 (4.2)	10 (7.5)	1.79 (0.73-4.36)	0.203
Histology	Intestinal	127 (33.7)	94 (36.3)	1.07 (0.73-1.55)	0.733	16 (4.2)	18 (6.9)	1.82 (0.83-3.99)	0.136
	Diffuse	127 (33.7)	49 (33.1)	0.92 (0.61-1.41)	0.712	16 (4.2)	4 (2.7)	0.77 (0.25-2.40)	0.648

SNP, single nucleotide polymorphism; PVT1, Plasmacytoma variant translocation 1; GC, gastric cancer; CON, control; AOR, adjusted odds ratio. CI, confidence interval; LNM, lymph node metastasis.

[a] Adjusted for age and gender. $^{*}P < 0.05$.
