# Supplementary material for: The association between polymorphism of the long noncoding RNA, Plasmacytoma variant translocation 1, and the risk of gastric cancer
Source: Medicine (Baltimore). 2021 Dec 3;100(48):e27773. doi: 10.1097/MD.0000000000027773 (PMC9191314; doi:10.1097/MD.0000000000027773)
Supplement: Supplemental Digital Content [file medi-100-e27773-s002.pdf]

Table S2 Stratified analysis of rs13255292 SNP of *PVT1* in GC patients and controls by age and other clinical features

Age (yr)	Features		Dominant model (CC/CT+TT)				Recessive model (CC+CT/TT)			
			CON, N	GC, N	AOR (95% CI)	P^a	CON, N	GC, N	AOR (95% CI)	P^a
< 60	T	T1+T2	72 (36.9)	58 (41.7)	1.13 (0.66-1.93)	0.664	10 (5.1)	4 (2.9)	0.57 (0.13-2.48)	0.452
		T3+T4	72 (36.9)	20 (34.5)	0.81 (0.42-1.59)	0.547	10 (5.1)	3 (5.2)	1.31 (0.31-5.52)	0.711
	LNM	Positive	72 (36.9)	23 (34.8)	0.78 (0.41-1.47)	0.443	10 (5.1)	2 (3.0)	0.83 (0.16-4.27)	0.821
		Negative	72 (36.9)	55 (42.0)	1.22 (0.70-2.11)	0.485	10 (5.1)	5 (3.8)	0.80 (0.20-3.15)	0.751
	Stage	I+II	72 (36.9)	62 (40.8)	1.13 (0.66-1.91)	0.660	10 (5.1)	5 (3.3)	0.68 (0.17-2.68)	0.582
		III	72 (36.9)	16 (35.6)	0.84 (0.41-1.72)	0.641	10 (5.1)	2 (4.4)	1.11 (0.22-5.63)	0.897
	Histology	Intestinal	72 (36.9)	37 (39.8)	0.79 (0.42-1.49)	0.468	10 (5.1)	5 (5.4)	1.16 (0.28-4.76)	0.838
		Diffuse	72 (36.9)	31 (38.3)	1.09 (0.61-1.96)	0.776	10 (5.1)	1 (1.2)	0.41 (0.05-3.47)	0.415
≥ 60	T	T1+T2	55 (30.2)	60 (37.3)	1.33 (0.82-2.15)	0.248	6 (3.3)	8 (5.0)	1.98 (0.63-6.18)	0.241
		T3+T4	55 (30.2)	32 (30.8)	0.90 (0.50-1.59)	0.705	6 (3.3)	9 (8.7)	2.48 (0.79-7.75)	0.118
	LNM	Positive	55 (30.2)	33 (29.2)	0.79 (0.44-1.39)	0.404	6 (3.3)	8 (7.1)	1.80 (0.56-5.79)	0.322
		Negative	55 (30.2)	59 (38.8)	1.46 (0.90-2.37)	0.128	6 (3.3)	9 (5.9)	2.38 (0.79-7.19)	0.126
	Stage	I+II	55 (30.2)	65 (36.9)	1.33 (0.83-2.12)	0.241	6 (3.3)	9 (5.1)	2.03 (0.67-6.12)	0.208
		III	55 (30.2)	27 (30.3)	0.83 (0.45-1.52)	0.537	6 (3.3)	8 (9.0)	2.39 (0.74-7.71)	0.146
	Histology	Intestinal	55 (30.2)	57 (34.3)	1.12 (0.69-1.84)	0.642	6 (3.3)	13 (7.8)	2.63 (0.90-7.71)	0.077
		Diffuse	55 (30.2)	18 (26.9)	0.77 (0.40-1.48)	0.438	6 (3.3)	3 (4.5)	1.41 (0.33-5.99)	0.644

SNP, single nucleotide polymorphism; PVT1, Plasmacytoma variant translocation 1; GC, gastric cancer; CON, control; AOR, adjusted odds ratio. CI, confidence interval; LNM, lymph node metastasis. [a] Adjusted for age and gender. $^*P < 0.05$.
