# Supplementary material for: The association between polymorphism of the long noncoding RNA, Plasmacytoma variant translocation 1, and the risk of gastric cancer
Source: Medicine (Baltimore). 2021 Dec 3;100(48):e27773. doi: 10.1097/MD.0000000000027773 (PMC9191314; doi:10.1097/MD.0000000000027773)
Supplement: Supplemental Digital Content [file medi-100-e27773-s003.pdf]

Table S3 Stratified analysis of rs2608053 SNP of *PVT1* in GC patients and control by age and other clinical features

Age (yr)	Features		Dominant model (CC/CT+TT)				Recessive model (CC+CT/TT)			
			CON, N	GC, N	AOR (95% CI)	*P* [a]	CON, N	GC, N	AOR (95% CI)	*P* [a]
< 60	T	T1+T2	87 (44.4)	59 (42.5)	0.98 (0.58-1.65)	0.928	13 (6.6)	9 (6.5)	0.91 (0.33-2.57)	0.864
		T3+T4	87 (44.4)	16 (27.6)	0.51 (0.26-1.02)	0.055	13 (6.6)	2 (3.4)	0.40 (0.08-1.96)	0.257
	LNM	Positive	87 (44.4)	24 (36.4)	0.75 (0.40-1.39)	0.363	13 (6.6)	5 (7.6)	0.98 (0.31-3.11)	0.968
		Negative	87 (44.4)	51 (38.9)	0.85 (0.50-1.47)	0.568	13 (6.6)	6 (4.6)	0.58 (0.18-1.86)	0.355
	Stage	I+II	87 (44.4)	63 (41.4)	0.90 (0.54-1.51)	0.685	13 (6.6)	10 (6.6)	0.87 (0.32-2.41)	0.791
		III	87 (44.4)	12 (26.6)	0.49 (0.23-1.03)	0.061	13 (6.6)	1 (2.2)	0.27 (0.03-2.19)	0.217
	Histology	Intestinal	87 (44.4)	34 (36.6)	0.83 (0.45-1.55)	0.563	13 (6.6)	4 (4.3)	0.55 (0.15-2.06)	0.377
		Diffuse	87 (44.4)	31 (38.3)	0.76 (0.43-1.36)	0.361	13 (6.6)	5 (6.2)	0.85 (0.27-2.74)	0.791
≥ 60	T	T1+T2	88 (48.6)	86 (53.4)	1.32 (0.83-2.08)	0.241	16 (8.8)	21 (13.0)	1.28 (0.62-2.68)	0.506
		T3+T4	88 (48.6)	39 (37.5)	0.71 (0.41-1.21)	0.204	16 (8.8)	11 (10.6)	0.89 (0.37-2.15)	0.794
	LNM	Positive	88 (48.6)	43 (38.1)	0.73 (0.43-1.24)	0.242	16 (8.8)	13 (11.5)	1.07 (0.46-2.48)	0.874
		Negative	88 (48.6)	82 (53.9)	1.34 (0.84-2.13)	0.222	16 (8.8)	19 (12.5)	1.15 (0.54-2.45)	0.717
	Stage	I+II	88 (48.6)	93 (52.8)	1.26 (0.81-1.97)	0.310	16 (8.8)	21 (11.9)	1.17 (0.56-2.43)	0.685
		III	88 (48.6)	32 (36.0)	0.69 (0.39-1.22)	0.202	16 (8.8)	11 (12.4)	1.09 (0.45-2.64)	0.850
	Histology	Intestinal	88 (48.6)	80 (48.2)	1.10 (0.69-1.75)	0.701	16 (8.8)	24 (14.5)	1.35 (0.65-2.81)	0.416
		Diffuse	88 (48.6)	33 (49.3)	1.17 (0.65-2.11)	0.596	16 (8.8)	7 (10.5)	1.02 (0.39-2.71)	0.966

SNP, single nucleotide polymorphism; PVT1, Plasmacytoma variant translocation 1; GC, gastric cancer; CON, control; AOR, adjusted odds ratio. CI, confidence interval; LNM, lymph node metastasis.

[a] Adjusted for age and gender. $^{*}P < 0.05$.
